# Estimating the Effective Sample Size of Tree Topologies from Bayesian Phylogenetic Analyses

**DOI:** 10.1093/gbe/evw171

**Published:** 2016-07-19

**Authors:** Robert Lanfear, Xia Hua, Dan L. Warren

**Affiliations:** ^1^Department of Biological Sciences, Macquarie University, Sydney, Australia; ^2^Ecology, Evolution, and Genetics, Australian National University, Canberra, Australia

**Keywords:** tree distance, phylogenetics, topology, MCMC, ESS, phylogenomics

## Abstract

Bayesian phylogenetic analyses estimate posterior distributions of phylogenetic tree topologies and other parameters using Markov chain Monte Carlo (MCMC) methods. Before making inferences from these distributions, it is important to assess their adequacy. To this end, the effective sample size (ESS) estimates how many truly independent samples of a given parameter the output of the MCMC represents. The ESS of a parameter is frequently much lower than the number of samples taken from the MCMC because sequential samples from the chain can be non-independent due to autocorrelation. Typically, phylogeneticists use a rule of thumb that the ESS of all parameters should be greater than 200. However, we have no method to calculate an ESS of tree topology samples, despite the fact that the tree topology is often the parameter of primary interest and is almost always central to the estimation of other parameters. That is, we lack a method to determine whether we have adequately sampled one of the most important parameters in our analyses. In this study, we address this problem by developing methods to estimate the ESS for tree topologies. We combine these methods with two new diagnostic plots for assessing posterior samples of tree topologies, and compare their performance on simulated and empirical data sets. Combined, the methods we present provide new ways to assess the mixing and convergence of phylogenetic tree topologies in Bayesian MCMC analyses.

## Introduction

Many areas of biology rely on Bayesian estimates of phylogenetic trees. Almost all modern phylogenetic studies include a Bayesian analysis of the data, and other areas such as phylogeography, phylodynamics, and comparative studies rely on Bayesian estimates of phylogenetic trees even if the tree itself is not of primary interest (Pagel et al. 2004; Lemey et al. 2009; Volz et al. 2013; Aberer et al. 2015; Whidden and Matsen 2015). Because Bayesian estimates of phylogenies are so widely used, it is important that we can assess their reliability.

Bayesian estimates of phylogenetic trees take the form of a posterior distribution, which is typically a collection of around 1,000 phylogenetic trees that describes the uncertainty about the evolutionary relationships among a set of sequences. Posterior distributions of trees are sampled using Markov chain Monte Carlo (MCMC) methods, in which an algorithm explores the space of all possible phylogenetic trees, periodically recording the trees it encounters (Nylander et al. 2008; Ronquist et al. 2012; Aberer et al. 2014; Bouckaert et al. 2014). A key consideration when using MCMC methods is to determine whether the chain has been run for long enough, and whether enough samples have been taken, to reliably estimate the posterior distributions of the parameters of interest.

The effective sample size (ESS) is a useful tool for assessing the adequacy of posterior samples taken from an MCMC analysis (Drummond et al. 2006; Kuhner 2009; Rambaut et al. 2012; Ronquist et al. 2012). The ESS is a measure of the number of uncorrelated samples that would be needed to estimate the posterior distribution of a given parameter with equivalent precision to the estimate obtained from the MCMC. The ESS can be much lower than the number of samples from the MCMC because sequential samples are often autocorrelated, i.e., the difference between sequential samples is smaller than the difference between truly independent samples. Autocorrelation is the rule rather than the exception in phylogenetic MCMC analyses, due to limits on the efficiency with which MCMC analyses can explore different values of parameters and different phylogenetic tree topologies. Because of this, it is standard practice to ensure that the ESS of every parameter is above some sensible threshold before making biological inferences from the results of an MCMC analysis.

Phylogeneticists have settled on the arbitrary but pragmatic rule of thumb that the ESS of all parameters should be at least 200 for the posterior distributions to be accurately inferred (Drummond et al. 2006). When the ESS of any parameter in the analysis is less than 200, researchers have a number of options: they can re-run the same analysis for longer; they can attempt to improve the way that the MCMC samples continuous parameters or phylogenetic tree topologies by adjusting proposal moves and/or using Metropolis Coupling (known as MCMCMC or MC^3^); and they can perform independent replicates of the same MCMC and combine the samples post-hoc. All of these approaches are sensible, but the current protocol has an important omission: we lack a method to calculate or estimate an ESS of the tree topologies sampled from the MCMC.

Tree topologies are arguably the most important parameter in phylogenetic MCMC analyses. The tree topology is the primary focus of many phylogenetic analyses, and regardless it can heavily influence the estimation of many other parameters such as substitution rates and divergence dates. On top of this, tree topologies are often expected to have a lower ESS than most continuous parameters; the number of possible topologies can be vast, and it is typically more difficult to sample from the set of all possible trees (described hereafter as tree space) than it is to sample from the set of all possible values of a standard continuous parameter (Huelsenbeck et al. 2002; Ronquist et al. 2012; Aberer et al. 2014; Bouckaert et al. 2014; Aberer et al. 2015; Whidden and Matsen 2015). This has the important ramification that an adequate ESS for all continuous parameters in an analysis cannot guarantee an adequate ESS of tree topologies from same analysis. Thus, there are few if any cases in which it would not be prudent to assess the adequacy of the sample of tree topologies from a Bayesian phylogenetic analysis before making biological inferences from the tree topology or any other parameters of interest (Kass et al. 1998).

Some methods already exist to examine posterior distributions of tree topologies, such as measuring trends in the distributions of split frequencies or the posterior distribution of trees in tree space. For example, the software AWTY (Nylander et al. 2008) allows users to analyse the stability of posterior support for splits within and between chains, and MrBayes calculates the average standard deviation of split frequencies between independent chains (Ronquist et al. 2012). Both of these methods can provide useful information on the stationarity and convergence of chains, but neither addresses the question of the sample size of tree topologies. Similarly, plotting trees in tree space (Hillis et al. 2005; Whidden and Matsen 2015) can provide useful visual information on MCMC performance and may provide visual clues to autocorrelation, but does not allow users to estimate an ESS of the tree topologies. Quantitative estimates of an ESS of tree topologies would be useful additions to existing methods of assessing MCMC performance. They would provide a convenient ways to assess the adequacy of posterior samples of trees from empirical analyses, and would be useful for comparing methods of exploring tree space, because all else being equal we should prefer methods that generate larger ESS values.

In this article, we present approaches to assess the autocorrelation and estimate an ESS of tree topologies from Bayesian phylogenetic MCMC analyses. We demonstrate and test the methods on a collection of empirical and simulated data sets, and show how they might be useful to users and developers of Bayesian phylogenetic MCMC methods.

## Materials and Methods

We present two new ways to visualise samples of tree topologies from Bayesian MCMC analyses, and two new ways to estimate an ESS of these samples.

### Visualization 1: Topology Traces: Visually Assessing the Progress of an MCMC in Tree Space

Visual inspection of parameter traces is a simple and widely used way of assessing MCMC performance. For a standard continuous parameter, a parameter trace shows the value of each sample of a given parameter on the *Y*-axis against the generation of the MCMC at which that sample was taken on the *X*-axis. A well-behaved MCMC (i.e., one with good mixing and little autocorrelation) will produce a parameter trace in which the value of the parameter appears to be sampled from a stationary distribution (i.e., the trace shows no long-term trends or large and sustained changes in value), and in which sequential samples are no more similar to each other than distantly related samples. Furthermore, parameter traces from replicate analyses should show qualitatively similar traces.

Parameter traces can be particularly useful in diagnosing problems with MCMC performance. Autocorrelation can be revealed by a trace that moves slowly around the stationary distribution. Poor mixing can be revealed by long-term trends in a parameter trace, or by a trace that spends a large number of generations around one value, before making a large jump and spending a large number of generations at another value.

**Fig. 1.—:**
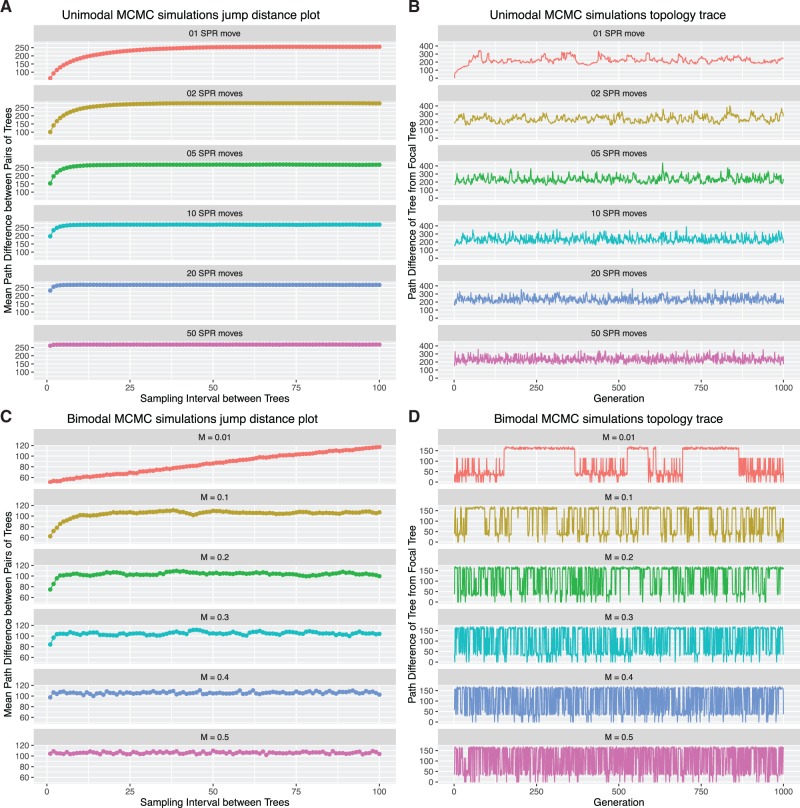
Jump distance plots and topology traces for twelve simulated posterior samples of trees. The plots demonstrate that as mixing improves (top to bottom panel in each sub-plot) autocorrelation decreases. In A and B, each data set contains 1,000 trees of 50 taxa, generated by iteratively applying a fixed number of SPR moves to a random starting tree. The number of SPR moves between trees in the data set is given in the title bar of each sub-plot. In *C* and *D*, each data sets contains 1,000 trees of 50 taxa, generated by sampling from two distantly-related sets of trees, where there is a probability M of switching between sets at each generation. The probability M is shown in the title bar of each sub-plot.

We propose a simple method for making traces of tree topologies that are analogous to traces for continuous parameters. In a topology trace, the *Y*-axis shows the topological distance of each sampled tree from a single focal tree, and the *X*-axis shows the generation at which each sample was taken (e.g. fig. 1B and D). For the assessment of multiple replicate analyses we suggest that a single focal tree topology is used, such that a when two replicate chains visit the same tree topology, they will show the same value on the *Y*-axis. This has the advantage that topology traces from replicate analyses that have converged to sample from the same stationary distribution should all show similar values on the *Y*-axis.

Topology traces have one limitation when compared with traces of continuous parameters—more than one tree topology in the posterior sample may share a single phylogenetic distance from any given focal tree. For example, if an MCMC spent the first half of the analysis sampling one tree topology, and the second half of the analysis sampling a very different topology, a topology trace could appear completely flat if the focal tree was equidistant from the two sampled trees. This situation is probably very rare in practice, but it does mean that topology traces could produce occasional false-negatives (i.e., they may fail to identify problematic analyses), although we do not expect them to produce false positives (i.e., to falsely identify as problematic an analysis that was not). These issues could be avoided by plotting multiple topology traces with different starting trees. Nevertheless, analysing topology traces together with traces of other parameters may provide useful insights into MCMC performance.

### Visualization 2: Jump Distance Plots: Visually Assessing Autocorrelation between Tree Topologies from MCMC Analyses

All else being equal, higher autocorrelation results in lower ESS values for a given parameter. An MCMC in which certain parameters show high autocorrelation might need to be run for longer to achieve a sufficient ESS for that parameter, and/or improvements might need to be made to the way that the MCMC explores parameter space to reduce autocorrelation. The degree of autocorrelation for a parameter can be expressed as the autocorrelation time, which is defined as the minimum interval required to ensure that sequential samples of that parameter are uncorrelated. At the limit, an autocorrelation time of 1 implies no autocorrelation for that parameter. Higher autocorrelation times indicate more severe autocorrelation. For continuous parameters, the autocorrelation time can be calculated with standard methods (George 1994; Kass et al. 1998). However, phylogenetic tree topologies are not continuous parameters, and existing analytical methods cannot be applied.

Here, we suggest that the autocorrelation of tree topologies from an MCMC analysis can be assessed by measuring the phylogenetic distances between pairs of trees at increasing sampling intervals (i.e., jump distances): an approach that for convenience we call a jump distance plot. If sequential samples are uncorrelated, the phylogenetic distance between pairs of trees will be unrelated to the sampling interval. In this case, the jump distance plot will be approximately flat, because the mean phylogenetic distance between pairs of trees will not depend on the sampling interval. However, if sequential samples are autocorrelated, then pairs of trees from smaller sampling intervals will tend to be more similar to each other than pairs of trees from larger sampling intervals. That is, the phylogenetic distance between pairs of trees will increase as the sampling interval increases, giving a jump distance plot with a positive slope. In the presence of autocorrelation, the jump distance plot may reach an asymptote when the sampling interval is large enough that pairs of trees effectively independent. In this case, the sampling interval at which the asymptote is reached provides an estimate of the autocorrelation time for tree topologies sampled from that MCMC. Of course, if the autocorrelation is severe relative to the length of the MCMC, then the jump distance plot might not reach an asymptote. In this case, the plot can only be used to place a lower bound on the autocorrelation time (i.e., it must be larger than the largest sampling interval in the plot), rather than a point estimate. This reasoning assumes that one is analysing samples from a well-mixed chain. In the absence of good mixing, autocorrelation plots (and thus the approximate-ESS, see below) may be misled. For this reason, we recommend assessing mixing (e.g., using a topology trace) before making inferences from a jump distance plot or the approximate-ESS (see below).

We have written R code to produce jump distance plots. In this code, we first load a posterior sample of *N* trees using ape’s read.tree function (Paradis et al. 2004). We then define a vector of sampling intervals, *s*, from 1 to [*N*/10], i.e., the largest sampling interval is the largest integer not greater than *N*/10. Note that these numbers correspond to intervals between sampled trees, rather than intervals in terms of MCMC generations. If the vector of sampling intervals is longer than 100, we subsample it to generate exactly 100 sampling intervals. This ensures that the data are easy to visualise, and allows us to work efficiently with large data sets. Next, for each sampling interval, *s_i_*, we calculate a vector of phylogenetic distances, *d_i_*, between overlapping pairs of trees from the posterior sample. For example, for *s_2 _*= 2, the first three pairs of trees would comprise the first three overlapping pairs of trees that are two trees apart in the posterior sample, i.e., the three pairs would, respectively, have the indices: (1, 3), (2, 4), and (3, 5). This vector of distances, *d_i_*, is our estimate of the distribution of phylogenetic distances at sampling interval *i*, which we summarise by taking the arithmetic mean. We calculate phylogenetic distances using the phangorn package in R (Schliep 2011). Finally, we visualise these data using the ggplot2 library in R (Wickham 2009).

We compare two methods of measuring the phylogenetic distance between trees: the Path Difference (Steel and Penny 1993) and the Robison–Foulds distance (Robinson and Foulds 1981; Hein et al. 1996). Many other such metrics exist, including the Subtree-Prune and Regraft distance (SPR distance; Steel and Penny 1993; Hein et al. 1996; Whidden et al. 2010) and the Nearest-Neighbor Interchange distance (NNI distance; DasGupta et al. 2000), both of which calculate differences between pairs of tree topologies while ignoring branch lengths (which we ignore because they are continuous parameters that can be assessed with conventional methods). We chose the Path Difference and the Robinson–Foulds distance because both are relatively fast to calculate and are implemented in R.

When autocorrelation is present, we expect all topological distance metrics to show patterns of increasing distance among pairs of trees sampled at increasing intervals. However, we do not expect all distance metrics to give quantitatively identical results. For example, the Robinson–Foulds metric can take a maximum value between quite similar trees, and has a relatively small number of possible values compared with the number of possible pairs of trees (Steel and Penny 1993). The Path Difference does not suffer from these limitations to the same extent: it has a larger range and better power to discriminate pairs of trees (Steel and Penny 1993). Thus, although the Robinson–Foulds metric is much faster to calculate, we expect the Path Difference to be more useful for estimating an ESS of tree topologies. More generally, we expect the best distance metrics for investigating autocorrelation and an ESS of tree topologies to be those with the best power to discriminate among unique pairs of tree topologies.

### Method 1: The Pseudo-ESS of Tree Topologies from Phylogenetic MCMC Analyses

Conventional methods for calculating the ESS are restricted to continuous parameters, and cannot be applied directly to phylogenetic tree topologies. However, it has been suggested before (Alexei Drummond, personal communication) that an ESS of tree topologies could be estimated by first converting the topologies into a continuous parameter, and then calculating an ESS with standard methods.

We estimate an ESS of tree topologies by randomly selecting a tree from the posterior sample as a focal tree, and then calculating the topological distance of every tree in the chain from that focal tree, as is visualised in the topology trace (fig. 1). This creates a vector of distances in which the focal tree has a distance of zero, and all trees that differ topologically from the focal tree have positive non-zero distances. We use this vector to calculate an ESS with standard approaches (see below). Thus, this method equates to treating the topology trace (Visualization 1, above) exactly as one would treat the trace of a continuous parameter.

We implement this approach in R, calculating vectors of Path Differences and Robinson-Foulds distances between the focal tree and all other trees using the phangorn package, as above. We then calculate an ESS of these vectors using the coda package (Plummer et al. 2006). We note that we cannot guarantee that this approach will yield the smallest ESS for a particular posterior sample of trees, and we therefore refer to it as a pseudo-ESS. Large pseudo-ESS values should be treated with caution, because there may be other statistics of tree topologies that would give smaller values. Furthermore, because the choice of the focal tree is arbitrary, we re-calculate the pseudo-ESS 100 times, randomly choosing a new focal tree each time. From these 100 replicates, we calculate the median and 95% confidence intervals of the pseudo-ESS for a given sample of phylogenetic tree topologies.

### Method 2: The Approximate-ESS of Tree Topologies from Phylogenetic MCMC Analyses

Standard methods to calculate the ESS divide the number of samples by the integrated autocorrelation time. We adapt these methods to derive an approximation of the ESS for phylogenetic tree topologies, by building on the approach we describe above for producing jump distance plots. Our derivation relies on an analogy between a topology and a continuous variable, such that the expected squared pairwise distances between samples of tree topologies is analogous to the covariance of samples of a continuous variable. This allows us to proceed with our derivation along the same lines as the classical approach for estimating ESS for continuous variable, where the ESS is the sample size of independent samples whose variance is equivalent to the covariance of the observed samples.

We first define the squared distance between two independent topologies from the posterior distribution, *D*, so the expected squared distance of *M* independent samples is $\frac{M(M-1)}{4M^{2}}D$. Then we ask how many independent samples will have the equivalent expected squared distance of *N* topologies sampled from the posterior distribution. *M* can be solved by the equation:

$$\frac{M(M-1)}{4M^{2}}D=\frac{\Sigma_{i=1}^{N-1}\Sigma_{k=1}^{\min(m,N-i)}f(k)+\frac{\left(N-m+1)(N-m\right)}{2}D}{2N^{2}},$$

where in sequential samples of the *N* topologies, *f*(*k*) is the squared distance between two samples that at a sampling interval of *k*. *m* is the minimum sampling interval between two samples that are independent from each other, i.e., the sampling interval at which the asymptote on the jump distance plot is reached.

To solve for M, we need to estimate f(k) for each *k *= 1,… *m* − 1, by taking the average squared distance between two random samples that are separated by a sampling interval of *k*. We also need to estimate *D*, by taking the average of the squared distances between two random samples that are separated by a sampling interval of at least *m*.

We have implemented this approach in R, using both the squared Path Difference and the squared Robinson–Foulds distance as measures of topological distance. To do this, we calculate the mean squared distance between pairs of trees at the first 100 sampling intervals, as described above. We then use the optim() function in R to estimate a best-fit model for the sampling distance at which the asymptotic squared topological distance is reached. This model is based on the exponential semivariogram (Chilès and Delfiner 2009), and takes the form:

$$f(k)=D\left(1-e^{-\frac{k}{a}}\right)$$

where *a* is a parameter that controls the shape of the exponential function, and *k* and D are as described above. If the model suggests that an asymptote has not been reached within the range of sampling distances available, we calculate an upper bound on the ESS by assuming that *D* is the largest mean squared topological distance sampled.

### Simulated and Empirical Data sets for Testing the Methods

We assess the methods we present here using simulated and empirical data sets. We do not know of a way to simulate phylogenetic MCMC data sets with a known ESS, so instead we simulated a large collection of MCMC data sets in which we expect the ESS to vary predictably (see below). We also apply our methods to six empirical data sets from a recent study of Malagasy herpetofauna (Scantlebury 2013a). We chose these data sets because the posterior samples of phylogenetic trees were publically available on DataDryad (Scantlebury 2013b), and because all of the analyses were carried out with rigorous and identical methods. These included: (i) running each analysis in BEAST v1.6.1 (Drummond and Rambaut 2007) for 20,000,000 generations, sampling trees every 10,000 generations; (ii) assessing stationarity and convergence of continuous parameters using TRACER (Rambaut et al. 2012); and (iii) assessing stationarity and convergence of tree topologies using AWTY (Nylander et al. 2008). In the original study, the author also repeated each analysis four times. For simplicity, we use only a single replicate of each analysis in this study.

### Analysis 1: Comparing Jump Distance Plots, Topology Traces, and ESS Estimates for 12 Simulated MCMC Data sets

In this analysis, we simulated 12 posterior samples of trees from unimodal and bimodal posterior distributions. We later use a much larger set of simulations to compare the estimates of the ESS in more detail. The simulations are designed to vary the degree of mixing from very poor to almost perfect. We expect the ESS to be low when mixing is poor, and increase to approach the number of trees in the sample (1,000 in this case) as mixing improves.

For the unimodal simulations, we created posterior samples of 1,000 trees by picking a random starting tree of 50 taxa, and generated subsequent samples by making a predetermined number of SPR moves on the previous sample using the rSPR function from the phangorn package (Schliep 2011). This is roughly equivalent to performing a phylogenetic MCMC without any sequence data. The larger the number of SPR moves between samples the more efficient the mixing, because the more independent sequential samples will be from one another. We simulated the output of six MCMC analyses with 1, 2, 5, 10, 20, and 50 SPR moves between sequential samples.

For the bimodal simulations, we first simulated two distantly related sets of 10 trees of 50 taxa. To do this, we seeded the first set with a random tree of 50 taxa, and then applied 50 random SPR moves to this tree to create the first tree of the second set. We simulated the subsequent nine trees of each set by applying one random SPR move to the starting tree of each set. Empirically, a posterior distribution of this type may be encountered if two loci with different evolutionary histories are concatenated into a single alignment. For each simulated MCMC, we created posterior samples of 1,000 trees in which the first tree is randomly chosen from set 1, and subsequent trees are chosen from either set 1 or set 2. The degree of mixing is controlled by a single parameter*, M*, which defines the probability that the current tree is chosen from the same set as the previous tree. When *M = *0.5 mixing is perfect because each generation has an equal probability of sampling a tree from either of the two sets. As *M* decreases from 0.5 towards zero mixing gets progressively worse, because the MCMC becomes more likely to take the sequential samples from the same set of trees. When *M* is exactly zero mixing is indistinguishable from perfect, because the MCMC will exclusively sample trees at random from set 1, and provide no evidence in the posterior sample of trees that set 2 exists. We simulated the output of six MCMC analyses of 50 taxa with value of *M* of 0.01, 0.1, 0.2, 0.3, 0.4, and 0.5. We thus expect the ESS to increase monotonically as *M* increases.

For each of the 12 simulations, we produced a topology trace and an jump distance plot, and calculated the approximate-ESS and the pseudo-ESS with 100 replicates as described above, using Path Differences as our measure of topological distance.

### Analysis 2: Comparing Jump Distance Plots, Topology Traces, and ESS Estimates for Six Empirical Data sets

For each of the six empirical data sets, we produced a topology trace and a jump distance plot, and calculated the approximate-ESS and the pseudo-ESS with 100 replicates as described above, using Path Differences as our measure of topological distance.

### Analysis 3: Assessing the Performance of the Pseudo-ESS and the Approximate-ESS Across a Wide Range of Simulated MCMC Data sets

For this analysis, we simulated unimodal and bimodal posterior distributions of trees as in analysis 1, but across a much wider range of parameter values, and using two different topological distance metrics: the Path Difference (as above) and the Robinson–Foulds distance.

For the unimodal simulations, we simulated 600 posterior samples of 1,000 trees as in analysis 1, comprising one simulation at each of 600 combinations of parameter values: 5, 20, and 100 taxa combined with 1–200 SPR moves between samples. For the bimodal simulations, we simulated 101 posterior samples of 1,000 trees as in analysis 1, comprising one simulation at each value of *M* from 0 to 0.5 in increments of 0.005.

For each simulation, we calculated the approximate-ESS and the pseudo-ESS as described above. We repeated the calculations using both the Path Difference and the Robinson–Foulds distance as measures of topological distance. For clarity, we report only the median pseudo-ESS calculated from the 100 replicates.

## Results

### Analysis 1: Comparing Jump Distance Plots, Topology Traces, and ESS Estimates for Six Simulated Well-behaved MCMC Data sets

As expected, the jump distance plots show positive slopes when mixing is poor (fig. 1*A* and *C*, top panel). These plots also show that in most cases, the topological distance between pairs of trees increases to a stable asymptote. The sampling interval at which this asymptote is reached decreases as mixing improves (i.e., as you move from the top to the bottom panels in fig. 1*A* and *C*), approaching a value of 1 when mixing is very efficient (bottom panels of fig. 1*A* and *C*).

The topology traces reveal useful details of the analysis. For example, topology traces from the unimodal simulations (fig. 1*D*) clearly show that the similarity of sequential samples decreases as mixing improves (i.e., as you move from the top to the bottom panel of fig. 1*D*). The topology traces from the bimodal simulations reveal the frequency with which the MCMC moves between the two different sets of trees. Within each set of simulations, the six topology traces reveal that the six analyses had converged to sample from similar regions of tree space, revealed by similar values of the topological distances on the *Y*-axis in the six sub-panels of fig. 1*B* and *D*, respectively.

The topology traces reveal a visual shortcoming of the approach: in the top panel of figure 1*B*, the trace starts from zero and rises quickly until it approaches a topological distance of ∼250. A standard interpretation of this trace for a continuous parameter would be to interpret the first 100 samples as sampling different parameter values than subsequent samples. However, in this case, the effect is an artefact: it results from the choosing the first tree of the simulated MCMC as the focal tree form which all other tree distances in figure 1*B* were calculated. This might be avoided by using a randomly generated tree that was not sampled from the MCMC as a focal tree, or another tree such as a Neighbor-Joining or Maximum Likelihood tree.

**Fig. 2.—:**
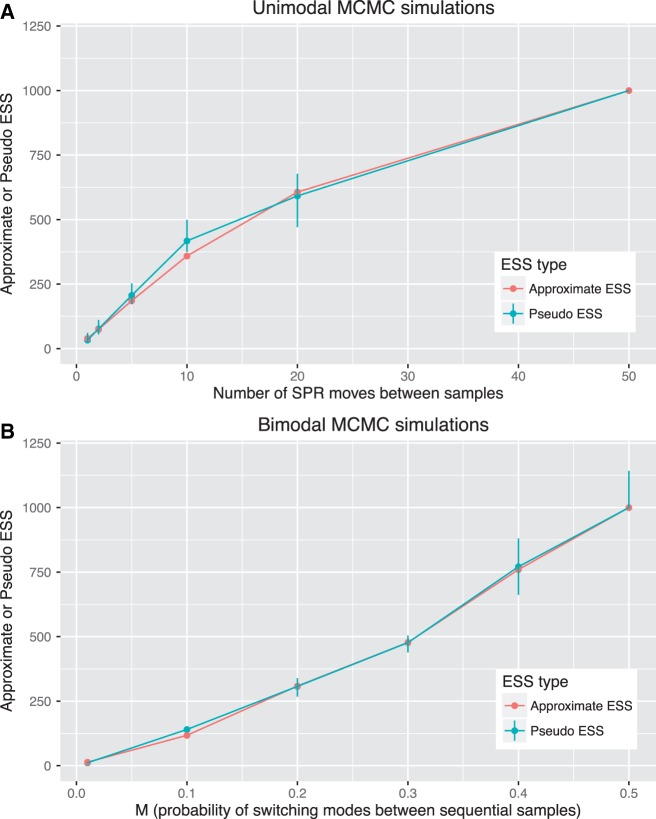
The approximate- and pseudo-ESS are very similar for twelve simulated posterior samples of trees. The data sets are the same as presented in figure 1. Both the approximate-ESS (pink dots) and the pseudo-ESS (blue dots, lines are 95% confidence intervals) rise to equal the number of trees in the chain (1,000) as mixing improves. Improved mixing is determined by the number of SPR moves between trees increasing (A) or by the probability of switching between sets of trees increasing (B). The agreement between the two estimates is striking.

The pseudo-ESS and the approximate-ESS of tree topologies are highly comparable for the twelve simulated data sets (figure 2). For both the unimodal and bimodal simulations, the value of the two ESS estimates increases as mixing improves, approaching a value equal to the length of the chain (i.e., 1,000) as mixing becomes very efficient.

### Analysis 2: Comparing Jump Distance Plots, Topology Traces, and ESS Estimates for Six Empirical Data sets

**Fig. 3.—:**
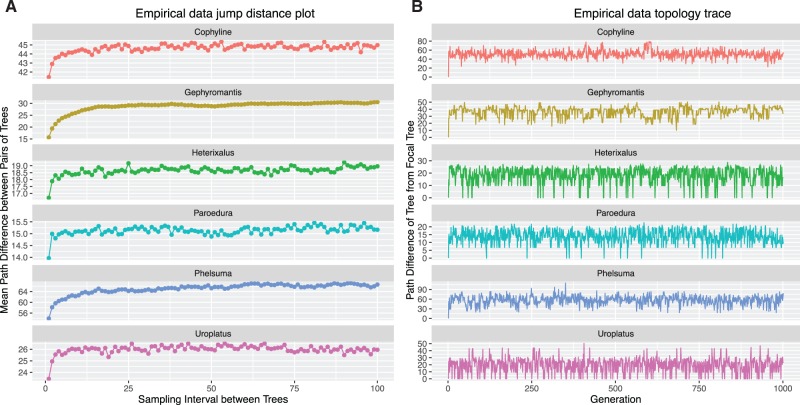
Jump distance plots and topology traces for six empirical posterior samples of trees. Each data set contains 1,000 phylogenetic trees generated by Bayesian MCMC analyses of DNA sequence alignments from Malagasy herpetofauna (Scantlebury 2013a). The figure shows that some data sets (e.g., *Gephyromantis*) show substantial autocorrelation in the posterior sample of trees, whereas others (e.g., *Paroedura*) show very little autocorrelation.

Figure 3 reveals autocorrelation among tree topologies in all six empirical data sets. In every case, the jump distance plot shows that the mean Path Difference between pairs of trees increases with the sampling interval. The amount of autocorrelation appears to be negligible for at least three data sets (*Heterixalus, Paroedura*, and *Uroplatus*), for all of which the mean distance between pairs of trees quickly reaches an asymptote (fig. 3*A*). The plots reveal significant autocorrelation in the remaining three data sets—the jump distance plot reaches an asymptote at sampling intervals larger than 10 for the *Cophyline, Gephyromantis*, and *Phelsuma* data sets (fig. 3*A*). These patterns are reflected in the topology traces, which show periods in each of these three analyses in which the Path Difference (*Y*-axis) stays relatively constant for >100 generations, suggesting that the MCMC was sampling similar tree topologies for extended periods (fig. 3*B*).

**Fig. 4.—:**
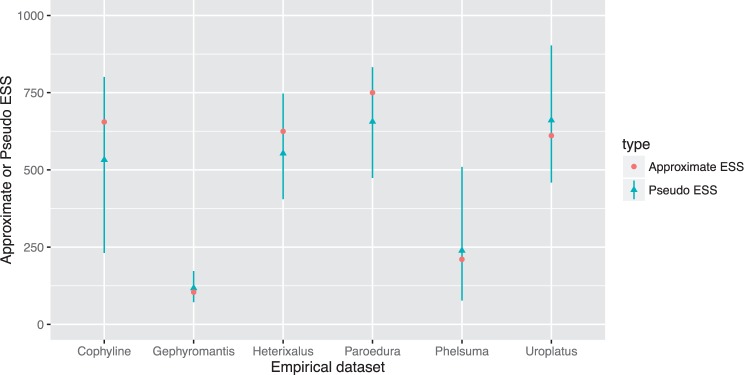
The approximate- and pseudo-ESS are very similar for six empirical posterior samples of trees. Each data set contains 1,000 phylogenetic trees generated by Bayesian MCMC analyses of DNA sequence alignments from Malagasy herpetofauna (Scantlebury 2013a). Within each data set, the approximate-ESS (pink dots) and the pseudo-ESS (blue dots, lines are 95% confidence intervals) agree closely, but there is substantial variation between data sets despite the fact that the MCMC parameters were identical in each case.

The approximate-ESS falls within the 95% confidence intervals of the pseudo-ESS for all six empirical data sets (fig. 4). Both the pseudo- and approximate-ESS values for the *Gephyromantis* data set fall well below the widely used cutoff of 200 (e.g., the approximate-ESS is 104), suggesting that the samples from this MCMC may not be sufficient to reliably estimate a posterior distribution of phylogenetic trees. The ESS values for the *Phelsuma* data set are also very close to the cutoff of 200 (e.g., approximate-ESS of 211) suggesting that the posterior distribution of trees from this MCMC may also benefit from further sampling.

### Analysis 3: Assessing the Performance of the Pseudo-ESS and the Approximate-ESS Across a Wide Range of Simulated MCMC Data sets

**Fig. 5.—:**
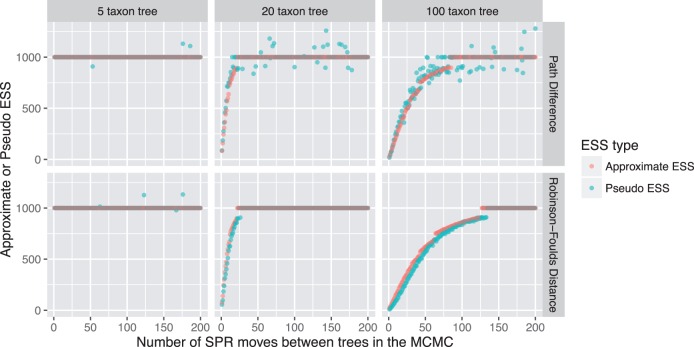
Approximate- and pseudo-ESS calculated with different distance metrics for 600 posterior samples of trees. Each data set contains 1,000 trees, generated by iteratively applying a fixed number of SPR moves to a random starting tree. The number of taxa in the tree is shown in the different columns, and the results of calculating ESS estimates with different distance metrics is shown in the different rows. Each plot shows the approximate-ESS (pink dots) and the median pseudo-ESS (blue dots) with a given number of SPR moves between trees in the MCMC. Both estimates agree closely, and both depend on the number of taxa in the tree, the distance metric used, and the number of SPR moves between trees in the MCMC.

The 600 simulations of unimodal posterior simulations (fig. 5) reveal that all four estimates of the ESS we compared (both the pseudo- and the approximate-ESS calculated with the Path Difference and the Robinson–Foulds distance) behave as expected across a wide range of parameter values. First, all estimates approach an asymptote of the length of the simulated chains (1,000) as the number of SPR moves between samples in the simulation increases. The pseudo-ESS (blue dots, fig. 5) but not the approximate-ESS (pink dots, fig. 5) occasionally gives an estimated value >1,000, an effect that is more pronounced when using the Path Difference rather than the Robinson–Foulds distance. Second, the rate of increase in all estimates of the ESS is lower when there are more taxa in the tree. For example, with a 5-taxon tree (fig. 5, left-hand column) almost every ESS estimate is 1,000, i.e., the asymptote is reached with just 1 SPR move between sequential samples in the simulated MCMC. With a 20 taxon tree (middle column, fig. 5), the ESS increases rapidly until it reaches an asymptote at around 50 SPR moves. And, with a 100 taxon tree (right-hand column, fig. 5), the ESS increases more slowly until it reaches an asymptote at around 100–150 SPR moves between sequential samples in the simulated MCMC.

**Fig. 6.—:**
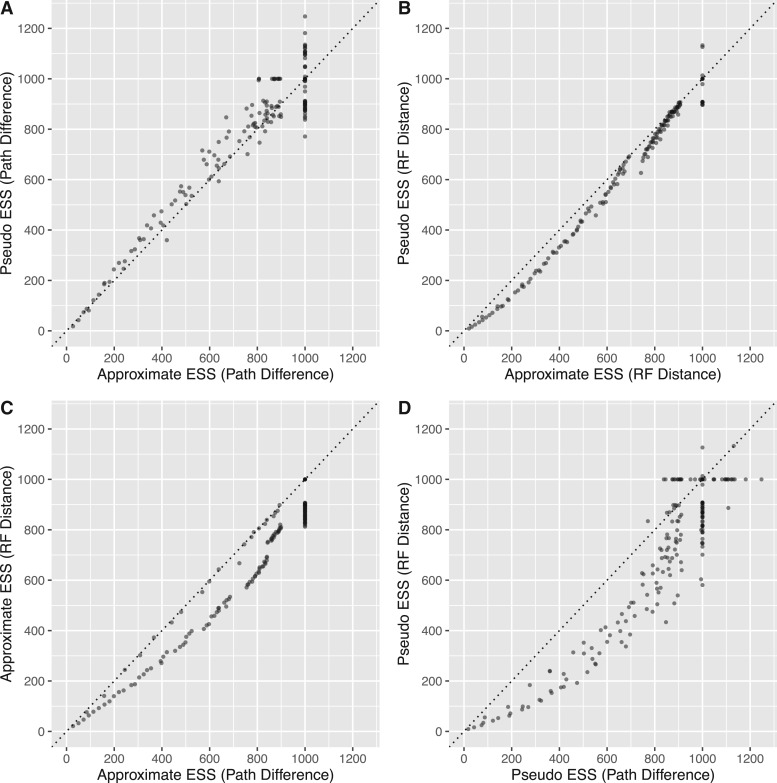
Comparison of the approximate- and pseudo-ESS calculated with different distance metrics for 600 posterior samples of trees. This figure shows a different view of the data from the 600 simulations presented in figure 5. Each dot represents a single simulated data set, and the axes of each sub-plot show two different estimates of the ESS for that data set. A and B compare the approximate-ESS (*X*-axis) to the pseudo-ESS (*Y*-axis) when calculated with the Path Difference and Robinson–Foulds distance, respectively. C and D compare the use of the Path Difference (*X*-axis) to the Robinson–Foulds distance (*Y*-axis) when used to calculate with the approximate-ESS and the pseudo-ESS, respectively. The dotted line in each plot represents a 1:1 relationship on which the points would lie if the two estimates agreed precisely.

Figure 6 reveals some differences in the four methods for estimating an ESS of tree topologies. First, when the ESS is estimated using the Path Difference (fig. 6*A*), the approximate-ESS gives a slightly smaller estimates than the pseudo-ESS. This trend is reversed when the ESS is estimated using the Robinson–Foulds distance (fig. 6*B*). Both the approximate-ESS (fig. 6*C*) and the pseudo-ESS (fig. 6*D*) give larger estimates when calculated with the Path Difference rather than the Robinson–Foulds distance.

**Fig. 7.—:**
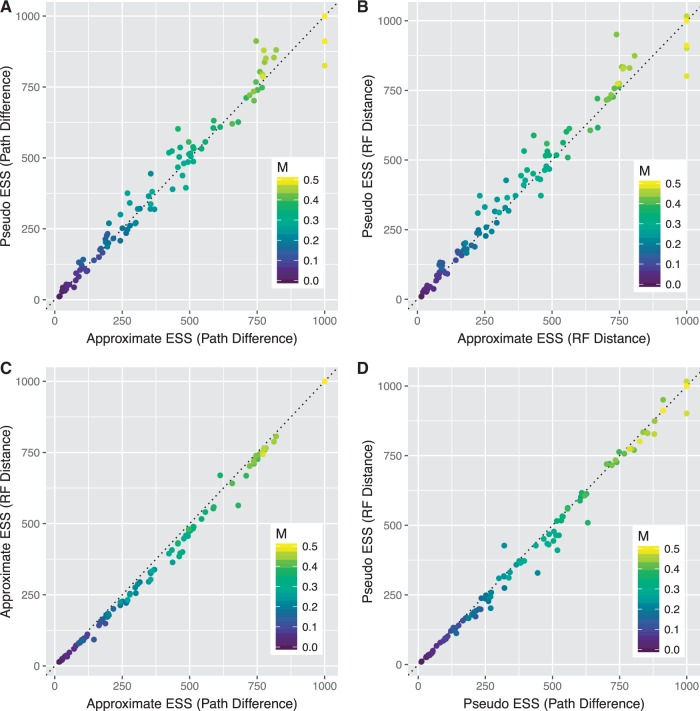
Comparison of the approximate- and pseudo-ESS calculated with different distance metrics for 101 posterior samples of trees. Each data set contains 1,000 trees, generated by sampling from two distantly related sets of trees, where there is a probability M (shown in the colour legend of each plot) of switching between sets at each generation. A and B compare the approximate-ESS (*X*-axis) to the pseudo-ESS (*Y*-axis) when calculated with the Path Difference and Robinson–Foulds distance, respectively. C and D compare the use of the Path Difference (*X*-axis) to the Robinson–Foulds distance (*Y*-axis) when used to calculate with the approximate-ESS and the pseudo-ESS, respectively. The dotted line in each plot represents a 1:1 relationship on which the points would lie if the two estimates agreed precisely.

All four methods of estimating an ESS of tree topologies are highly comparable for the simulations of bimodal posterior samples of trees (fig. 7). As expected, all estimates of the ESS increase as the degree of mixing increases (blue to yellow colour change, fig. 7; note that when M = 0, all methods returned an ESS of 1,000 as expected, though this is not visible from fig. 7 due to overplotting). The differences between the pseudo- and the approximate-ESS do not appear to be biased (fig. 7*A* and *B*, respectively). There is a slight tendency for ESS values estimated with Path Differences to be larger than those calculated with Robinson–Foulds distances (fig. 7*C* and *D*), as with the unimodal simulations.

Figures 5–7 reveal one limitation of the approximate-ESS of tree topologies: it is somewhat granular when the ESS is close to the number of posterior samples (1,000 in this case). This is shown most clearly in figure 5, where the approximate-ESS (pink dots) does not increase smoothly with the number of SPR moves. This occurs because the calculation of the approximate-ESS relies on calculating the sampling interval between trees at which the asymptote in the jump distance plot is reached (fig. 1*A*). This creates granularity because the sampling interval is an integer. For example, when mixing is very good this sampling interval will be 1 and the ESS will equal the number of samples taken from the chain. As mixing becomes worse the sampling interval will remain at 1 until the mixing is poor enough that the estimated sampling interval is 2, this change precipitates the jumps in the approximate-ESS that can be seen clearly in the right-hand column of figure 5. Our formulation of the approximate-ESS does not allow us to use non-integer sampling intervals in the calculation, so this shortcoming would be difficult to rectify in the current framework. However, we do not expect this limitation to be of practical importance, because the aim is to diagnose cases where the ESS is low (e.g., <200) and the number of posterior samples is usually far higher than this (e.g., at least ∼1,000).

## Discussion

We present four new methods for assessing the adequacy of tree topologies sampled from Bayesian MCMC analyses: topology traces, jump distance plots, the pseudo-ESS, and the approximate-ESS. We demonstrate and test the methods with simulated and empirical data sets. Here, we compare and discuss the methods, and provide practical guidelines for their application to empirical data.

Topology traces and jump distance plots provide convenient and complementary methods for visually assessing posterior samples of tree topologies. The ideal posterior sample of tree topologies has good mixing and consequently little or no autocorrelation. This produces a relatively flat jump distance plot and a topology trace without any visible long-term trends, as in the lowest panels of figures 1*A*–*D*, which were generated from simulations with very good mixing. Poor mixing creates autocorrelation, reflected by rising jump distance plots and topology traces which show sustained sampling from similar trees, as in the top panels of figures 1*A*–*D*. Topology traces also allow quick visual assessment of convergence. If all of the topology traces from replicate analyses have similar values on the *Y*-axis, this suggests (but does not quite guarantee, see above) that all of the analyses were sampling trees from the same region of tree space (fig. 1*B*).

Surprisingly, we detected autocorrelation in all of the empirical studies we analysed here (fig. 3*A*). This is despite these MCMCs having been run for 20,000,000 generations, with samples collected at large intervals of 10,000 generations—parameters that would usually be considered more than adequate for a Bayesian phylogenetic analysis. This is more notable because the number of taxa in these analyses was relatively small (from 9 to 61), and the trees were inferred with simple models of molecular evolution (a single GTR + G model applied to each data set). These conditions should be optimal for minimising autocorrelation among tree topologies in a Bayesian phylogenetic analysis: fewer taxa means that there are exponentially fewer possible trees; and simpler models means that the MCMC can spend proportionally more time proposing changes to the phylogeny. This suggests that autocorrelation among tree topologies in Bayesian phylogenetic analyses may be the rule rather than the exception. It highlights the importance of assessing tree topologies sampled from Bayesian MCMC analyses, particularly as new software and new data sets lead to larger and more complex analyses (Faircloth et al. 2012; Lemmon et al. 2012; Ronquist et al. 2012; Aberer et al. 2014; Bouckaert et al. 2014).

The pseudo- and approximate-ESS provide convenient ways to quantify the adequacy of posterior samples of tree topologies derived from Bayesian MCMC analyses. Both measures estimate of the number of independent samples of tree topologies represented by the output of a phylogenetic MCMC analysis, and provide similar estimates over a wide range of simulated and empirical data sets (figs. 2 and 4–7). The lowest ESS estimate for tree topologies that we detected in an empirical data set here was 104 (*Gephyromantis*, figs. 3 and 4). This is well below what might be considered sufficient to derive an accurate estimate of the posterior distribution of trees. In this case, the low ESS is unlikely to have affected the conclusions of the original study (Scantlebury 2013a), because the author ran four independent analyses and combined the results, likely increasing the total approximate-ESS to ∼400. Nevertheless, these low values highlight the important impact that autocorrelation might have on the accuracy of phylogenies estimated from Bayesian MCMC analyses.

The pseudo- and approximate-ESS can produce systematically different estimates of the ESS on the same data (fig. 6*a* and *b*), and we prefer the approximate-ESS over the pseudo-ESS for a number of reasons. First the approximate-ESS is mathematically derived with an approach analogous to the calculation of the ESS for continuous parameters. This makes its assumptions explicit, and provides a solid basis for further development. Second the approximate-ESS does not require the arbitrary selection of a focal tree. We know of no basis on which to prefer a single focal tree for calculating the pseudo-ESS, and our analyses show that the value of the pseudo-ESS can vary substantially depending on which focal tree is chosen. For example, in one empirical data set (*Phelsuma*, fig. 4), when the pseudo-ESS was recalculated with 100 randomly selected focal trees, the lowest estimate was more than an order of magnitude smaller than the largest estimate (fig. 4 shows the 95% confidence intervals). The utility of an estimate that can have such drastic uncertainty is questionable when a rigorously derived point estimate (i.e., the approximate-ESS) exists. Despite our preference for the approximate-ESS, the similarity, in most cases, of the two ESS estimates derived here is notable.

Our results demonstrate that different topological distance measures can produce substantially different ESS estimates (fig. 5*C* and *D*). The Path Difference tends to give larger estimates of the ESS than the Robinson–Foulds distance for a given data set (fig. 5*C* and *D*) in almost all of the conditions we simulated. We suspect that the reason for this discrepancy is that the Path Difference is more discriminating than the Robinson–Foulds distance: the Robinson–Foulds distance has a relatively small number of unique values for a given collection of pairs of trees, whereas the Path Difference has a much larger number of unique values (Steel and Penny 1993). This is particularly severe when the trees being compared are highly dissimilar, because the Robinson–Foulds metric also has a relatively small maximum value (Steel and Penny 1993). The more often unique pairs of trees have the same difference for a given metric, the more that metric is likely to overestimate the autocorrelation between samples, and thus underestimate the ESS. We should therefore prefer estimates of the topological ESS calculated with distance metrics that are the most discriminating, i.e., those that tend to assign unique values to the differences between unique pairs of tree topologies.

We suggest that the most convenient way to use the methods we propose here is to examine the topology trace and the approximate-ESS of tree topologies for each phylogenetic MCMC. The topology trace allows for rapid visual assessment of the posterior sample of trees, and provides a way to quickly assess whether the chain is well mixed. If the chain is well mixed, it is appropriate to use the approximate-ESS to quantify the adequacy of the posterior sample. To this end, both of these methods can be invoked via a single command in the RWTY software [https://github.com/danlwarren/RWTY; the makeplot.topology() command].

It is interesting to consider extensions to the methods we propose here. First, although we provide two methods to estimate an ESS for tree topologies, a comprehensive solution to the problem of calculating the ESS remains open. Second, it may be possible to calculate or estimate an ESS of subsets of taxa that make up the tree. This could be achieved by extracting these subsets from each tree in the posterior sample, and applying the methods we describe above, or by breaking down the Path Difference into the contributions of each separate pair of taxa. This could facilitate identification of parts of the topology that have been problematic in an analysis. Third, by tracking an estimate of the ESS of tree topologies and other parameters during an MCMC, it may be possible to tune proposal moves so that the MCMC proposes moves to parameters in inverse proportion to their current ESS. This could help improve the efficiency of phylogenetic MCMC analyses by focussing the most effort on the parameters with the smallest ESS values.

The methods we propose join a growing collection of approaches for visually and quantitatively assessing phylogenetic tree topologies sampled from Bayesian MCMC analyses (Hillis et al. 2005; Nylander et al. 2008; Bouckaert 2010; Ronquist et al. 2012; Bouckaert et al. 2014; Whidden and Matsen 2015). They give us a new tools for comparing the merits of different topological proposal methods, calculating the appropriate balance between proposing moves on the tree topology versus other parameters, and determining how many MCMC samples are adequate to achieve biologically meaningful estimates of phylogenetic trees in a Bayesian framework.
